# Multivariate Temporal Inflammatory–Regenerative Signatures of Bovine Platelet-Rich Gel Supernatants Under Different Storage Temperatures

**DOI:** 10.3390/gels12050422

**Published:** 2026-05-12

**Authors:** Jorge U. Carmona, Catalina López

**Affiliations:** 1 Grupo de Investigación Terapia Regenerativa, Departamento de Salud Animal, Universidad de Caldas, Calle 65 No. 26-10, Manizales 170004, Colombia; 2 Grupo de Investigación Patología Clínica Veterinaria, Departamento de Salud Animal, Universidad de Caldas, Calle 65 No. 26-10, Manizales 170004, Colombia

**Keywords:** fibrin matrix, biomaterials, controlled release, cytokines, growth factors, tissue repair, wound healing, regenerative therapy, veterinary medicine, cattle

## Abstract

Platelet-rich gel supernatants (PRGS) are increasingly used in veterinary medicine due to their regenerative and immunomodulatory properties; however, most studies focus on individual mediators and provide limited insight into their coordinated biological behavior. This study aimed to characterize the integrated inflammatory–regenerative signatures of bovine PRGS stored under different temperature conditions using a multivariate approach. Concentrations of transforming growth factor beta-1 (TGF-β1), tumor necrosis factor alpha (TNF-α), interleukin-2 (IL-2), and interleukin-6 (IL-6) were evaluated in PRGS samples from six clinically healthy cows stored at −80, −20, 4, 21, and 37 °C for up to 326 h. Data were standardized and explored using hierarchical clustering and heatmaps, and principal component analysis (PCA) based on area under the concentration–time curve (AUC) was used to integrate temporal behavior. Temperature-dependent multivariate signatures were identified, with frozen PRGS clustering separately from samples stored at moderate temperatures. The first two principal components explained 43.0% and 28.9% of the variance and defined an inflammatory–regenerative gradient contrasting TGF-β1/IL-2 versus TNF-α/IL-6 profiles. Linear mixed-effects modeling showed that PC1 was significantly affected by temperature and time (*p* < 0.001), whereas PC2 was influenced by temperature, time, and their interaction (*p* ≤ 0.048). Differences among temperatures were minimal at early time points but became more pronounced from 48 to 96 h onward, following a temperature gradient with higher values at moderate temperatures and lower values under frozen conditions. These findings indicate that storage temperature reshapes the integrated biological profile of PRGS, rather than merely preserving mediator composition.

## 1. Introduction

Platelet-rich plasma (PRP) and platelet-rich gel (PRG)-derived products have gained increasing attention in veterinary medicine due to their regenerative and immunomodulatory potential. These biological products contain a complex and dynamic mixture of growth factors (GF) and cytokines that collectively influence tissue repair, inflammation, and immune responses [1,2,3]. In bovine medicine, PRP, platelet-rich gel supernatants (PRGS), and related hemocomponents represent a practical therapeutic alternative, particularly under field conditions, where they have been used in the management of mastitis, reproductive disorders, wounds, and subsolar ulcers [4,5]. However, despite their apparent simplicity of preparation, one of the main challenges limiting their broader use is the preservation of biological activity during handling and storage.

Concerns regarding contamination, degradation, or denaturation of GF have constrained the implementation of standardized storage strategies. Notably, current clinical practice often relies on freshly prepared platelet concentrates administered shortly after processing, highlighting the limited understanding of how storage conditions influence the stability of PRGS bioactive profiles [4,6]. Previous experimental studies, including a work from our group [7], have demonstrated that both time and temperature significantly affect the release and stability of individual mediators in bovine PRGS, such as transforming growth factor beta-1 (TGF-β1) and several pro-inflammatory cytokines. These univariate approaches have been instrumental in identifying how specific molecules behave under different storage conditions. However, PRP and related hemocomponents do not exert their biological effects through isolated mediators. Instead, their bioactivity emerges from the coordinated and simultaneous action of multiple GFs and cytokines, functioning as an integrated biological system [3,8].

In this context, PRG can be understood as a fibrin-based biological matrix with hydrogel-like properties, capable of retaining and gradually releasing a complex mixture of bioactive mediators. The supernatant derived from PRG reflects the soluble fraction of this dynamic system, integrating signals that are relevant for inflammation and tissue repair. From a biomaterials perspective, such PRG-derived matrices share functional features with naturally derived hydrogels, in which environmental conditions may influence the balance and release patterns of embedded mediators. Understanding how storage conditions shape these integrated mediator profiles may therefore contribute to a broader interpretation of PRG as a biologically active matrix rather than a simple source of isolated mediators.

From this perspective, evaluating PRGS behavior mediator by mediator may overlook important system-level changes induced by storage conditions. Shifts in the relative balance between inflammatory and regenerative mediators may be more biologically relevant than absolute changes in individual concentrations, particularly in complex inflammatory systems where coordinated interactions determine functional outcomes [9,10,11]. Understanding PRGS as a coordinated multivariate system is therefore essential to better interpret how storage conditions shape its overall biological profile.

Multivariate analytical approaches provide a practical way to examine coordinated patterns among platelet-, leukocyte-, and matrix-associated mediators in PRGS, allowing a more integrated interpretation than individual mediator measurements [12]. Such integrative strategies have shown that preparation-related factors can reshape the overall biochemical composition of PRP beyond isolated mediator changes [13]. Despite their potential, multivariate analyses appear to remain uncommon in veterinary PRP-derived product research, where interpretation is still largely dominated by traditional, largely univariate reporting approaches, in a field that has long recognized platelets as complex regenerative and immunomodulatory players [14].

Building upon previously generated experimental data [7], the present study applies a multivariate approach to characterize inflammatory–regenerative signatures of bovine PRGS stored at different temperatures and times. Rather than re-evaluating individual mediator behavior, the study examines whether storage temperature and time are associated with coordinated multivariate profiles of PRGS. Hierarchical clustering and heatmap visualization were used to identify overall patterns, while principal component analysis (PCA) based on time-integrated mediator exposure was used to explore the main sources of variation while reducing pseudo-replication [15,16,17].

We hypothesized that storage temperature may be associated with differences in multivariate inflammatory–regenerative patterns in bovine PRGS that are not captured by univariate analyses of individual mediators. Frozen storage was hypothesized to be associated with relatively higher pro-inflammatory contributions, whereas moderate temperatures (21–37 °C) were hypothesized to be associated with more balanced regenerative–immunoregulatory profiles, with temperature acting as a primary factor shaping multivariate patterns. Accordingly, this study applies a multivariate approach to explore integrated PRGS signatures and to examine how these patterns vary in relation to storage temperature and time, without aiming to establish definitive inferential comparisons between storage conditions. Within this context, the aim was to examine whether PRGS exhibit coherent multivariate inflammatory–regenerative patterns associated with storage temperature, with potential relevance to veterinary clinical and field applications.

## 2. Results and Discussion

### 2.1. Multivariate Inflammatory–Regenerative Signatures of PRGS

**Figure 1:**
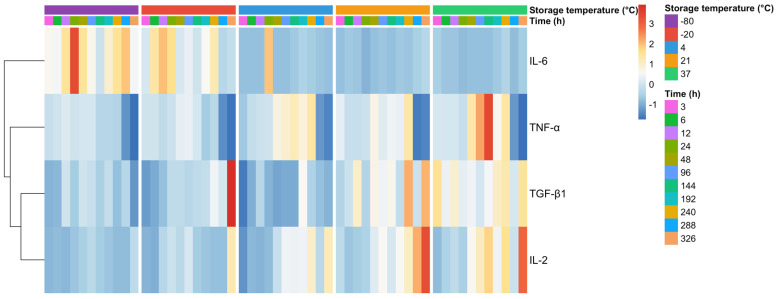
Heatmap of standardized (z-score) concentrations of inflammatory and regenerative mediators (TNF-α, IL-6, IL-2, and TGF-β1) in bovine PRGS across storage temperatures and time points. Values represent mean concentrations for each temperature × time combination. Z-scores were calculated per mediator to emphasize relative patterns. Columns are ordered by temperature and time, with gaps indicating transitions between temperature conditions. Colors represent deviations from the mediator-specific mean (blue: lower; red: higher), and the dendrogram shows hierarchical clustering of mediators based on their temporal–thermal profiles. Data are based on n = 6 animals.

Exploratory multivariate analysis revealed that storage temperature was the primary factor shaping the global inflammatory–regenerative profile of PRGS. Heatmap visualization based on standardized mediator values demonstrated distinct clustering patterns among temperature conditions, indicating the presence of coordinated multivariate signatures rather than isolated mediator-specific changes. The raw dataset underlying these analyses is available as Dataset S1 in the Supplementary Materials. Frozen storage conditions (−80 °C and −20 °C) consistently clustered separately from PRGS maintained at moderate temperatures (21 °C and 37 °C), whereas samples stored at 4 °C exhibited an intermediate profile (Figure 1).

Across temperature–time combinations, frozen PRGS showed relative enrichment of pro-inflammatory mediators, particularly IL-6, while PRGS stored at 21 °C and 37 °C displayed a more balanced inflammatory–regenerative profile characterized by higher relative contributions of TGF-β1 and IL-2. Although storage time influenced mediator behavior within each temperature condition, temporal effects were secondary to temperature in determining overall multivariate structure (Figure 1).

### 2.2. PCA of Time-Integrated Mediator Profiles

**Figure 2:**
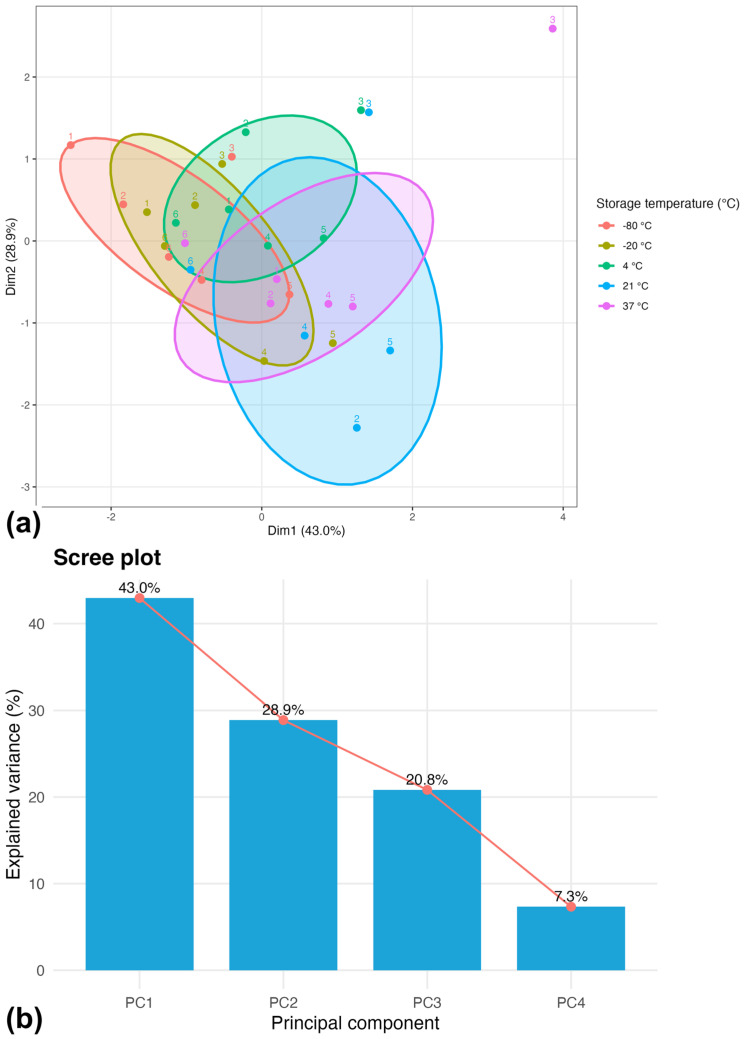
Principal component analysis (PCA) of time-integrated mediator profiles based on AUC values for TNF-α, IL-6, IL-2, and TGF-β1. (**a**) PCA score plot showing each cow–temperature combination, with labels indicating individual animals. Colored ellipses represent the 68% confidence region for each storage temperature, illustrating dispersion and partial overlap among conditions. The first two components explained 43.0% (Dim1) and 28.9% (Dim2) of the total variance. (**b**) Scree plot showing the proportion of variance explained by each component. All groups included n = 6 animals after exclusion of non-finite AUC values.

Principal component analysis revealed a clear temperature-associated multivariate structure of PRGS profiles based on AUC-integrated mediator data. The first two principal components explained a substantial proportion of the total variance (Dim1: 43.0%; Dim2: 28.9%), indicating that the dominant sources of variation in integrated mediator exposure were captured by these components (Figure 2).

Dim1 represented the main inflammatory–regenerative gradient across storage conditions, separating frozen samples from those maintained at moderate temperatures. In contrast, Dim2 provided additional resolution within moderate-temperature conditions, particularly distinguishing PRGS stored at 21 °C and 37 °C (Figure 2a).

Within each temperature condition, AUC values exhibited moderate inter-individual variability, as reflected by the dispersion of observations within clusters. This variability remained within the overall temperature-associated multivariate structure. The scree plot further confirmed that most of the variance was explained by the first two principal components, supporting their use for subsequent analyses (Figure 2b).

### 2.3. Contribution of Individual Mediators to Principal Components

**Figure 3:**
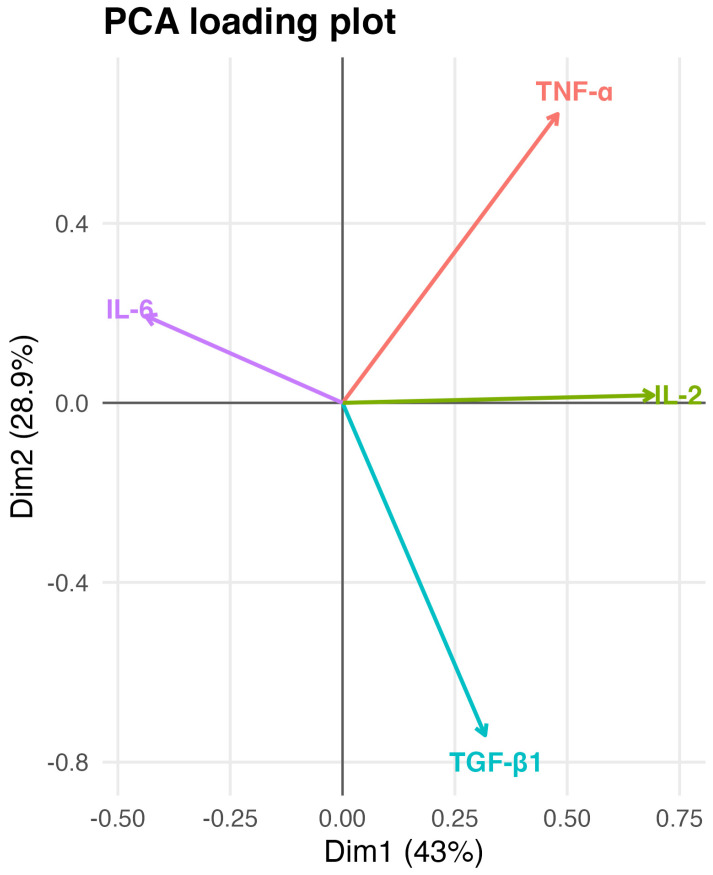
Loading plot of the principal component analysis showing the contribution and direction of each mediator to the first two principal components. Arrows represent the loadings of TNF-α, IL-6, IL-2, and TGF-β1 on Dim1 and Dim2, with arrow length indicating the magnitude of contribution and orientation reflecting correlations among mediators. Dim1 is primarily associated with pro-inflammatory (TNF-α) and immunoregulatory (IL-2) components, whereas Dim2 captures contrasting contributions of IL-6 and TGF-β1, suggesting differential inflammatory versus regenerative influences. Data are based on samples obtained from n = 6 animals. This visualization facilitates biological interpretation of the multivariate structure underlying the PCA scores shown in Figure 2.

Analysis of PCA loadings indicated that IL-2 and TGF-β1 contributed strongly and positively to Dim1, whereas IL-6 loaded in the opposite direction (Figure 3). This pattern indicates that Dim1 represents a dominant regenerative/immunoregulatory versus pro-inflammatory axis underlying PRGS multivariate behavior. TNF-α contributed positively to Dim1 but to a lesser extent than IL-2 and TGF-β1, indicating a comparatively smaller contribution to the global profile (Figure 3). Dim2 was primarily influenced by TGF-β1 in opposition to TNF-α, providing additional resolution between PRGS maintained at 21 °C and 37 °C (Figure 3).

### 2.4. Time-Integrated Mediator Exposure Across Storage Temperatures

Descriptive statistics of time-integrated mediator exposure are summarized in Table 1. Across storage temperatures, TGF-β1 showed the highest AUC values, with greater magnitudes at moderate temperatures (21–37 °C) compared with frozen conditions, whereas IL-6 displayed progressively lower AUC values as storage temperature increased. TNF-α exhibited substantial variability across temperatures, with higher integrated exposure at 4 °C and 37 °C relative to frozen storage.

In contrast, IL-2 demonstrated increasing AUC values from frozen to moderate temperatures, followed by greater inter-individual variability at 37 °C. Although moderate variability among animals was observed within each temperature group, the overall patterns of time-integrated mediator exposure were consistent with the temperature-dependent multivariate structures identified by the heatmap and PCA analyses, providing quantitative context for the integrated inflammatory–regenerative signatures of PRGS. Absolute concentration profiles over time and across storage temperatures, including individual data points, are provided in Figures S1–S4 in the Supplementary Materials to facilitate direct visualization of the underlying data prior to time integration.

### 2.5. Association of Multivariate Structure with Storage Temperature and Time

Linear mixed-effects modeling of PCA scores showed that PC1 was significantly affected by storage temperature (F = 16.81, *p* < 0.001) and time (F = 4.66, *p* < 0.001), whereas the interaction between temperature and time was not significant (F = 0.90, *p* = 0.647). In contrast, PC2 was significantly affected by temperature (F = 11.45, *p* < 0.001), time (F = 2.88, *p* = 0.002), and their interaction (F = 1.44, *p* = 0.048) (Table 2).

**Figure 4:**
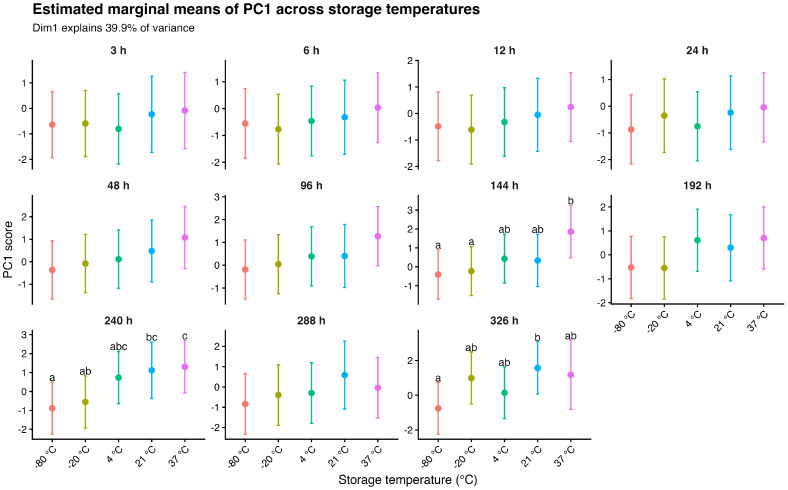
Estimated marginal means of PC1 derived from linear mixed-effects models across storage temperatures and time points. Values are presented with 95% confidence intervals. Panels correspond to individual time points (3–326 h), and colors indicate storage temperature conditions (−80 °C, −20 °C, 4 °C, 21 °C, and 37 °C). Different letters denote statistically significant differences among temperatures within each time point based on Holm-adjusted pairwise comparisons (*p* < 0.05). PC1 (Dim1) explains 43% of the total variance.

Estimated marginal means showed that differences among storage temperatures were minimal at early time points (3–24 h), where PC1 scores largely overlapped across conditions (Figure 4). From 48 to 96 h onward, a gradual separation emerged, becoming more evident at later stages (144–326 h). At these later time points, PC1 values followed a clear temperature gradient, with higher scores observed at moderate temperatures (21–37 °C), intermediate values at 4 °C, and lower scores under frozen conditions (−20 °C and −80 °C). This progressive divergence indicates a consistent temporal amplification of temperature-associated multivariate structure, with similar directional trajectories across conditions.

**Figure 5:**
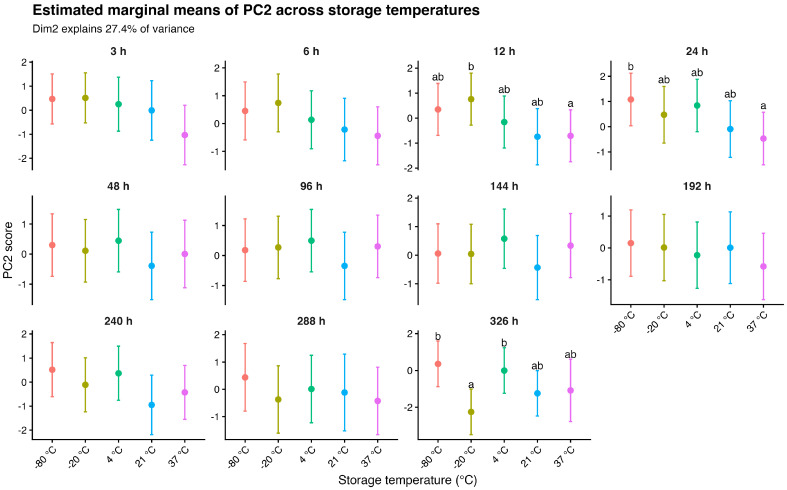
Estimated marginal means of PC2 derived from linear mixed-effects models across storage temperatures and time points. Values are presented with 95% confidence intervals. Panels correspond to individual time points (3–326 h), and colors indicate storage temperature conditions (−80 °C, −20 °C, 4 °C, 21 °C, and 37 °C). Different letters denote statistically significant differences among temperatures within each time point based on Holm-adjusted pairwise comparisons (*p* < 0.05). PC2 (Dim2) explains 27.4% of the total variance.

In contrast, PC2 exhibited a less uniform temporal pattern, with temperature-related differences appearing intermittently rather than progressively (Figure 5). While early time points showed substantial overlap among conditions, distinct separation emerged at specific time points (i.e., 12–24 h and 326 h), where individual temperature groups diverged in opposite directions. This irregular pattern, together with the presence of statistically distinguishable groups at selected time points, is consistent with the significant temperature × time interaction observed for PC2. Overall, these results indicate that PC1 captures a stable, temperature-dependent multivariate gradient that strengthens over time, whereas PC2 reflects more transient and condition-specific temporal dynamics.

Bootstrap resampling showed that the proportion of variance explained by PC1 and PC2 remained within comparable ranges across resampled datasets, with mean values of 47.8% and 26.1%, respectively, and percentile intervals of 38.0–69.3% for PC1 and 17.6–31.5% for PC2. The relative contribution of individual mediators to the principal components was preserved across bootstrap iterations, with high average correlations between resampled and original loading structures for both PC1 (r = 0.847) and PC2 (r = 0.818).

Leave-one-animal-out (LODO) analysis showed that sequential exclusion of individual animals resulted in only minor variation in explained variance, with mean values of 42.8% for PC1 and 27.5% for PC2. The loading structure remained largely consistent across exclusions, with correlations relative to the full dataset averaging 0.933 for PC1 and 0.839 for PC2. Overall, these results indicate that the identified multivariate patterns were not driven by a single individual and were preserved across resampling conditions.

To our knowledge, previous studies in bovine systems, and more broadly across veterinary and human species, have not explicitly evaluated the effects of storage conditions on PRP or related hemocomponents using a multivariate inflammatory–regenerative framework. By shifting the analytical focus from individual mediator measurements to integrated multivariate signatures, the present study provides a systems-level perspective on the behavior of bovine PRGS under different storage conditions. Although earlier investigations, including our own [7,18], have shown that storage temperature and time influence the concentration and stability of specific growth factors and cytokines, our findings suggest that these effects are more accurately interpreted as coordinated shifts in inflammatory–regenerative balance rather than as independent molecular alterations.

Heatmap analysis indicated that storage temperature was the primary factor shaping the multivariate profiles of PRGS, with frozen conditions (−80 °C and −20 °C) consistently clustering apart from samples stored at moderate temperatures (21 °C and 37 °C). This temperature-driven structuring is consistent with previous evidence showing that storage conditions promote coordinated biochemical and functional remodeling of platelet-derived products, affecting platelet activation, mitochondrial function, lipid composition, and the stability or release of growth factors and cytokines [19,20,21,22]. Proteomic and lipidomic studies further support the notion that storage induces system-wide molecular reorganization rather than isolated changes in individual components [20,23].

Building on this evidence, the present findings indicate that storage conditions actively reshape the biological state of PRGS rather than merely preserving it. Freezing was associated with a distinct shift toward profiles characterized by a relative predominance of pro-inflammatory components, suggesting a reconfiguration of the underlying mediator balance. In contrast, PRGS maintained at moderate temperatures (21 °C and 37 °C) retained more balanced multivariate profiles, consistent with preservation of coordinated regenerative and immunoregulatory signaling. The close concordance between hierarchical clustering and PCA further supports the robustness of this temperature-dependent reorganization of PRGS functional identity.

PRGS exhibited a dominant temperature-dependent shift in the balance between regenerative and immunoregulatory mediators (TGF-β1 and IL-2) and pro-inflammatory mediators, particularly IL-6, reflecting an underlying inflammatory–regenerative continuum captured by the PCA. This dominant pattern accounted for a substantial proportion of the overall variability and consistently differentiated storage conditions, supporting the concept that PRGS behavior is governed by an integrated mediator balance rather than by isolated molecular changes. Accordingly, these findings are more consistent with a continuous inflammatory–regenerative gradient than with a strict dichotomous classification, highlighting coordinated mediator contributions across a biological spectrum. Although IL-2 is traditionally associated with T-cell proliferation and may exert pro-inflammatory effects depending on the context, its contribution here is interpreted within a broader immunoregulatory and homeostatic framework, consistent with its role in regulatory T-cell maintenance and immune balance [24].

Importantly, linear mixed-effects modeling of PCA scores provided inferential support for the multivariate structure. Temperature was the main factor associated with variation in PC1, whereas PC2 reflected additional temperature-dependent temporal dynamics. The absence of a significant interaction for PC1, together with a significant interaction for PC2, supports the interpretation of a dominant temperature-driven gradient accompanied by more specific time-dependent modulation of multivariate profiles.

The biological relevance of this multivariate organization is supported by previous functional studies demonstrating that PRGS modulate inflammatory and regenerative responses through coordinated, time-dependent mediator release in target tissues. In in vitro systems of synovial membrane, cartilage, ligament, and tendon, PRGS induce concurrent changes in pro- and anti-inflammatory cytokines and GF, resulting in balanced immunomodulatory and anabolic responses [25]. At the cellular level, platelet-derived supernatants and lysates have also been shown to promote coordinated immunoregulatory phenotypes, including macrophage polarization toward reparative profiles characterized by suppression of pro-inflammatory mediators and activation of TGF-β–associated pathways [26]. These observations are consistent with the multivariate organization identified in the present study.

Importantly, previous experimental evidence further supports this interpretation [27]. PRP-derived products incubated at 37 °C, even in the presence of bacteria (conditions expected to accelerate proteolysis), have been shown to maintain relatively stable concentrations of key growth factors over time. These findings suggest that PRP-derived systems are more resilient than simple protein solutions and reinforce the concept that their behavior reflects a dynamic balance between degradation, sustained release, and matrix-associated protection, rather than true molecular preservation.

In vivo evidence further supports the concept that the bioactivity of PRP-derived products arises after platelet activation and fibrin polymerization within the tissue environment. Intramammary administration of fresh, calcium-activated P-PRP in bovine models has been shown to result in in situ formation of PRG, followed by the progressive release of PRGS into the mammary gland. This process leads to time-dependent modulation of multiple inflammatory and immunoregulatory mediators in milk, including IL-1, IL-2, IL-6, IL-8, IFN-γ, and TNF-α, reflecting a dynamic balance between pro-inflammatory and regulatory signals rather than a uniform anti-inflammatory response [4,6]. These observations demonstrate that PRP-based therapies exert their biological effects through the coordinated release of PRGS within tissues, providing physiological context for the multivariate mediator signatures identified in the present study.

Within this framework, the present findings extend existing functional and in vivo evidence by indicating that pre-analytical handling, particularly storage temperature, reshapes the integrated inflammatory–regenerative signatures of PRGS prior to tissue exposure. Rather than acting as a biologically neutral preservation step, freezing is associated with a shift in the overall mediator balance toward profiles with a greater pro-inflammatory contribution, whereas PRGS maintained at moderate temperatures retain more balanced multivariate configurations. These observations suggest that storage conditions influence not only mediator stability but also the integrated biological state of PRGS, with potential relevance for downstream biological activity.

Additional support for a systems-level interpretation of platelet-derived products comes from studies on leukocyte-platelet rich fibrin (L-PRF), which similarly functions as a three-dimensional fibrin scaffold releasing biologically active supernatants over time. In bovine L-PRF membranes [28], GF release follows distinct temporal patterns influenced by physiological context, highlighting that platelet-derived biomaterials behave as dynamic biological systems characterized by coordinated, time-dependent mediator release. Although PRF studies have primarily focused on individual growth factors and have not examined inflammatory cytokines or multivariate signatures, they reinforce the concept that platelet-based products exert their effects through integrated supernatant dynamics rather than static molecular composition.

Collectively, these findings support a conceptual framework in which the bioactivity of PRP-derived products (whether generated in vitro, released in situ following PRP activation, or derived from fibrin-based matrices such as PRF) emerges from coordinated multivariate mediator behavior. The present study contributes to this framework by demonstrating that storage conditions can reshape inflammatory–regenerative signatures prior to tissue exposure, thereby potentially influencing the biological state of PRGS released from platelet-rich gels. This systems-level perspective underscores the limitations of traditional univariate analyses and the need to consider integrated mediator balance when optimizing the preparation, storage, and application of PRP and related hemocomponents.

By integrating mediator exposure over time, the present analysis captures the dominant longitudinal signal underlying PRGS behavior while minimizing the influence of repeated measurements at the individual level [15]. At the biological level, storage temperature emerges as the principal factor structuring global PRGS profiles, with temperature-driven differences outweighing within-condition temporal variability across the evaluated interval [16,17]. Although temporal integration may obscure short-term fluctuations, it provides a robust summary of cumulative mediator exposure and facilitates interpretation of global multivariate profiles under different storage conditions. The close concordance between heatmap clustering and PCA further supports that the identified patterns reflect stable, biologically meaningful organization rather than transient dynamics.

Importantly, this integrative approach was intended to summarize overall mediator exposure and characterize global inflammatory–regenerative structure, rather than to model fine-scale temporal trajectories. While mixed-effects longitudinal models are more appropriate for capturing detailed time-dependent dynamics, this represents a distinct analytical objective and an important direction for future research [29].

From a veterinary perspective, these findings have relevant practical implications. In clinical and field settings, PRP-derived products are frequently prepared and stored under non-ideal conditions, with storage decisions often driven by logistical considerations rather than biological criteria. Accumulating evidence indicates that storage temperature influences multiple interconnected aspects of platelet biology, including activation status, metabolic activity, vesicle release, and cytokine accumulation, suggesting that its effects extend beyond the stability of individual growth factors. Accordingly, preservation of the overall inflammatory–regenerative balance may be as important as maintaining absolute mediator concentrations when optimizing storage protocols for platelet-derived products. From a biomaterials perspective, these findings are also relevant to the design of platelet-derived hydrogel systems for wound healing, where spatiotemporal regulation of bioactive mediator release is critical for coordinating inflammation and tissue repair. In this context, storage conditions may influence not only the composition but also the functional release behavior of PRGS, with potential implications for their use as biologically active components in hydrogel-based wound dressing strategies [19,30,31]. These considerations may also extend to human regenerative medicine, where PRP-derived biomaterials are increasingly applied in translational settings and similar principles of storage-dependent functional modulation are likely to be relevant.

Beyond clinical applications, these findings have important implications for experimental and translational research, particularly in embryo culture systems and stem cell research, where platelet lysates and PRP-derived supernatants have been proposed as alternatives to fetal bovine serum [32,33,34,35]. In these contexts, PRP and related hemocomponents are valued for their capacity to support cell proliferation, survival, and differentiation under serum-free or xeno-reduced conditions. However, accumulating evidence indicates that freeze–thaw processing and storage actively reshape the combined cytokine and growth factor milieu of platelet-derived preparations, altering release dynamics and integrated composition even when selected mediators appear quantitatively preserved [5,9,36]. Such modifications suggest that frozen platelet lysates or PRGS represent biologically distinct environments that may differentially influence embryonic development or stem cell behavior. Accordingly, the present findings highlight that the suitability of platelet-derived products as serum substitutes should be evaluated not only based on individual GF concentrations, but also in terms of their integrated inflammatory–regenerative profiles, which may critically shape downstream cellular responses in sensitive in vitro systems.

This study has several limitations that should be acknowledged. First, the analysis was exploratory in nature and conducted on a limited number of animals, reflecting the inherent constraints of controlled veterinary experimental studies. While this restricts statistical power and generalizability, it allowed a controlled evaluation of multivariate structure under standardized experimental conditions. The stability of the PCA structure was supported by bootstrap resampling and leave-one-animal-out analyses, which showed consistent explained variance and loading patterns across iterations. These findings indicate that the identified multivariate structure was not driven by individual animals and remained stable despite the limited sample size.

Second, the mediator panel was intentionally limited to a small number of cytokines and GF representing distinct inflammatory, immunoregulatory, and regenerative functions. Although this reduced dimensionality constrains the breadth of biological coverage, it enabled focused exploration of integrated multivariate patterns consistent with the exploratory aims of the study. PCA stability was explored through bootstrap resampling and leave-one-animal-out analyses, both of which supported the consistency of the identified component structure. However, these procedures evaluate statistical robustness rather than biological functionality, and therefore the identified multivariate signatures still require external and functional validation.

Third, functional bioactivity assays were not performed. Therefore, the identified multivariate signatures should be interpreted as biochemical proxies of biological state rather than direct measures of therapeutic efficacy. In this context, the use of animal-level AUC summaries partially accounts for inter-individual variability, although future studies should explicitly model random effects within multivariate longitudinal frameworks and incorporate functional validation.

In addition, protein integrity was not evaluated by Western blotting or equivalent structural assays, sterility testing was not performed during the full incubation period, and protease activity was not quantified. Therefore, ELISA-derived mediator concentrations should be interpreted as immunoreactive biochemical measurements rather than direct evidence of intact protein structure or functional bioactivity. Future studies should include protein-integrity assays, recombinant cytokine degradation controls, microbiological monitoring, and protease activity measurements to better define the mechanisms underlying mediator persistence in PRGS.

## 3. Conclusions

Storage temperature is a dominant determinant of the integrated biological profile of bovine PRGS, not by altering individual mediators in isolation, but by reshaping coordinated multivariate signatures that reflect overall inflammatory–regenerative balance. Frozen storage conditions were associated with profiles showing a relative predominance of pro-inflammatory components, whereas PRGS maintained at moderate temperatures exhibited more balanced regenerative–immunoregulatory signatures. By adopting a multivariate, systems-level framework, this work extends previous univariate observations and underscores the limitations of interpreting platelet-derived products solely on the basis of individual mediator concentrations. The multivariate signatures identified here should therefore be regarded as hypothesis-generating biochemical correlates of inflammatory–regenerative behavior, requiring confirmation and functional validation in independent experimental systems.

## 4. Materials and Methods

This study was approved by the Animal Experimentation Committee of the Universidad de Caldas (Manizales, Colombia) and conducted in accordance with ARRIVE guidelines [37]. The heifers included in the study were housed at an institutional farm under the direct responsibility of the research team. All blood collection procedures were performed in compliance with institutional animal welfare guidelines and under protocols approved by the corresponding ethical review committee. Only clinically healthy animals were included.

### 4.1. Study Design and Experimental Framework

This study represents a secondary integrative analysis of previously generated experimental data obtained from bovine PRGS [7]. The original experimental design aimed to evaluate the effects of storage temperature and time on the release and stability of selected growth factors and cytokines. The present analysis addresses a distinct research question by focusing on the coordinated multivariate behavior of these mediators rather than on their individual dynamics.

The experimental procedures for PRGS preparation, storage conditions, and mediator quantification have been described in detail elsewhere and were conducted under controlled laboratory conditions [7,18]. Briefly, PRGS samples were obtained from 6 clinically healthy Blanco Oreji-Negro heifers with a mean age of 24 months (range: 16–30 months), and subjected to storage at five different temperatures (−80 °C, −20 °C, 4 °C, 21 °C, and 37 °C). Samples were analyzed at predefined time points (0, 3, 6, 24, 72, 168, 288, and 326 h post-activation), covering both short-term and prolonged storage conditions. At each time point, independent aliquots were collected for biochemical analysis.

### 4.2. Preparation of PRP and PRGS

PRP was obtained from whole blood collected aseptically from each animal using a standardized centrifugation protocol designed to produce a pure PRP (P-PRP) formulation [4,6,7,18]. This protocol resulted in a moderate enrichment of platelets relative to baseline whole blood values and a marked depletion of leukocytes. Platelet concentrations were approximately 1.5–2-fold higher than those measured in whole blood, while leukocyte counts were reduced by more than one order of magnitude, yielding a PRP preparation with minimal leukocyte contribution (P-PRP). Although platelet enrichment in this preparation is lower than that reported in some PRP classification systems, it is consistent with pure PRP (P-PRP) formulations commonly used in veterinary applications. This cellular composition was consistent across animals and ensured that downstream analyses primarily reflected platelet-derived biological activity.

PRG formation was induced by chemical activation of PRP with calcium gluconate. Specifically, a 10% calcium gluconate solution (9.3 mg/mL; Ropsohn Therapeutics Ltda^®^, Bogotá, Colombia) was added to PRP at a 9:1 ratio, triggering platelet activation, fibrin network formation, and mediator release [7,18]. After completion of clot formation, the gel was processed to PRGS, which were separated from the fibrin matrix by centrifugation. Immediately after collection, PRGS were divided into aliquots to minimize degradation associated with repeated freeze–thaw cycles and subsequently maintained under predefined temperature conditions until biochemical analyses were performed.

### 4.3. Quantification of Growth Factors and Cytokines

Concentrations of transforming growth factor beta-1 (TGF-β1), tumor necrosis factor alpha (TNF-α), interleukin-2 (IL-2), and interleukin-6 (IL-6) in platelet-rich gel supernatants were quantified by enzyme-linked immunosorbent assay (ELISA). TGF-β1 was measured using a DuoSet ELISA Development System (Human TGF-β1 DuoSet, DY240E; R&D Systems, Minneapolis, MN, USA), previously validated for bovine samples based on the high sequence homology between bovine and human TGF-β1 [38]. TNF-α, IL-2, and IL-6 were quantified using species-specific bovine DuoSet ELISA kits (DY2279, DY2465, and DY8190, respectively; R&D Systems, Minneapolis, MN, USA).

All determinations were performed in duplicate in accordance with the manufacturers’ instructions. For TGF-β1 quantification, samples were acid-activated and diluted prior to analysis following the recommended protocol. This procedure resulted in a final dilution factor of approximately 1:40. Optical density was measured at 450 nm using a microplate spectrophotometer, and mediator concentrations were derived by interpolation from analyte-specific standard curves constructed with the provided recombinant standards. Final concentrations were calculated by applying the corresponding dilution factor.

### 4.4. Data Preprocessing and Standardization

Raw concentration data were organized by animal, storage temperature, and time point. To facilitate integrative multivariate analysis and allow comparison among mediators with different concentration ranges, values were standardized using z-score transformation within each mediator. This approach emphasizes relative changes and coordinated patterns across mediators rather than absolute concentration differences.

### 4.5. Statistical Analysis

All data processing and statistical analyses were performed using R statistical software (v.4.5.2, R Foundation for Statistical Computing, Vienna, Austria). Data manipulation was conducted using the dplyr (v.1.2.1) and tidyr (v.1.3.2) packages, while multivariate analyses and visualizations were performed using the factoextra (v.1.0.7) and pheatmap packages (v.1.0.13). Hierarchical clustering and heatmap visualization were used to identify global inflammatory–regenerative signatures of PRGS across storage conditions. Heatmaps were constructed using standardized mediator values averaged for each temperature–time combination, and clustering was applied to both mediators and experimental conditions using Euclidean distance and complete linkage [17].

PCA was performed as an exploratory dimensionality-reduction technique on time-integrated mediator exposure to account for the longitudinal structure of the data and reduce pseudo-replication. For this purpose, the area under the concentration–time curve (AUC) was calculated for each mediator per animal and storage temperature using the trapezoidal rule [15,29], and the resulting matrix was standardized prior to PCA. PCA results were interpreted based on mediator loadings, and scores were visualized using biplots to examine relationships among observations and mediators [16].

To complement the AUC-based analysis and evaluate temporal variation in multivariate structure, PCA was also performed on the standardized longitudinal dataset. Scores from the first two principal components derived from this analysis were used for subsequent modeling.

The association between multivariate structure and experimental conditions was assessed using linear mixed-effects models, including storage temperature, time, and their interaction as fixed effects and animal identity as a random intercept to account for repeated measurements within individuals. Statistical significance for mixed-effects models was set at *p* < 0.05. Post hoc comparisons of estimated marginal means were performed using Holm-adjusted contrasts where appropriate.

The stability of PCA results was evaluated using bootstrap resampling at the animal level, whereby animals were sampled with replacement and PCA was recalculated in each iteration. The consistency of mediator loadings and explained variance across bootstrap samples was used to assess stability of the multivariate structure. Robustness was further examined using a leave-one-animal-out sensitivity analysis, in which PCA was recalculated after sequential exclusion of each animal, and resulting loadings and explained variance were compared with those obtained from the full dataset.

Finally, descriptive statistics of time-integrated mediator exposure (AUC) were calculated as mean and standard deviation for each mediator and storage temperature to provide biological context for the multivariate patterns, without aiming at inferential comparisons between conditions.

## Figures and Tables

**Table 1:** Descriptive statistics of time-integrated mediator exposure (area under the concentration–time curve, AUC) for TNF-α, IL-6, IL-2, and TGF-β1 in bovine platelet-rich gel supernatants stored at different temperatures.

Mediator	Temperature	n	AUC (Mean ± SD) (pg/mL)
IL-2	−80 °C	6	143,768.35 ± 70,060.31
	−20 °C	6	171,590.21 ± 90,900.85
	4 °C	6	224,699.89 ± 95,862.40
	21 °C	5	274,944.16 ± 118,308.53
	37 °C	6	265,534.63 ± 166,404.32
IL-6	−80 °C	6	43,900.92 ± 32,116.12
	−20 °C	6	35,238.97 ± 21,718.29
	4 °C	6	19,179.75 ± 7867.49
	21 °C	5	16,232.29 ± 3449.05
	37 °C	6	15,056.97 ± 4423.36
TGF-β1	−80 °C	6	3,695,330.70 ± 759,066.86
	−20 °C	6	4,361,612.48 ± 1,445,612.58
	4 °C	6	3,223,333.70 ± 504,072.74
	21 °C	5	5,324,490.93 ± 2,145,940.09
	37 °C	6	4,678,295.82 ± 1,050,169.86
TNF-α	−80 °C	6	219,378.70 ± 121,663.85
	−20 °C	6	233,289.18 ± 113,933.07
	4 °C	6	335,063.75 ± 228,388.99
	21 °C	5	282,985.50 ± 215,379.20
	37 °C	6	400,831.71 ± 370,390.86

Values are expressed as mean ± standard deviation and calculated across individual animals (n indicated for each temperature). These descriptive summaries complement the exploratory multivariate analyses and do not imply inferential comparisons between storage conditions.

**Table 2:** Effects of storage temperature and time on PCA scores.

PC	Effect	Sum Sq	Mean Sq	NumDF	DenDF	F Value	*p*-Value
PC1	Temperature	54.81	13.70	4	280.20	16.81	<0.001
	Time	38.01	3.80	10	280.20	4.66	<0.001
	Temperature × Time	29.33	0.73	40	279.97	0.90	0.647
PC2	Temperature	29.42	7.35	4	280.43	11.45	<0.001
	Time	18.47	1.85	10	280.43	2.88	0.002
	Temperature × Time	37.09	0.93	40	279.99	1.44	0.048

Results are from Type III analysis of variance derived from linear mixed-effects models (LMMs). PCA: principal component analysis; PC: principal component; NumDF: numerator degrees of freedom; DenDF: denominator degrees of freedom. Animal identity was included as a random effect to account for repeated measurements within individuals. *p*-values were obtained using Satterthwaite’s approximation.

## Data Availability

The raw data supporting the conclusions of this article will be made available by the authors on request.

## Supplementary Materials

The following supporting information can be downloaded at: https://www.mdpi.com/article/10.3390/gels12050422/s1, Dataset S1: Raw dataset (Excel file) containing cytokine concentrations and optical density values; Figure S1: Box-and-whisker plots of absolute TNF−α concentrations across storage temperatures and time points, including individual data points. Boxes represent the interquartile range (IQR), the central line indicates the median, whiskers extend to the minimum and maximum non-outlier values, and dots represent individual observations. Figure S2: Box-and-whisker plots of absolute TGF−β1 concentrations across storage temperatures and time points, including individual data points. Boxes represent the interquartile range (IQR), the central line indicates the median, whiskers extend to the minimum and maximum non-outlier values, and dots represent individual observations. Figure S3: Box-and-whisker plots of absolute IL−2 concentrations across storage temperatures and time points, including individual data points. Boxes represent the interquartile range (IQR), the central line indicates the median, whiskers extend to the minimum and maximum non-outlier values, and dots represent individual observations. Figure S4: Box-and-whisker plots of absolute IL−6 concentrations across storage temperatures and time points, including individual data points. Boxes represent the interquartile range (IQR), the central line indicates the median, whiskers extend to the minimum and maximum non-outlier values, and dots represent individual observations.

## Abbreviations

The following abbreviations are used in this manuscript:

AUC	Area under the concentration–time curve
GF	Growth factor
IL-2	Interleukin-2
IL-6	Interleukin-6
LMM	Linear mixed-effects model
LODO	Leave-one-animal-out
PCA	Principal component analysis
PC	Principal component
PRF	Platelet-rich fibrin
PRG	Platelet-rich gel
PRGS	Platelet-rich gel supernatants
PRP	Platelet-rich plasma
P-PRP	Pure platelet-rich plasma
TNF-α	Tumor necrosis factor alpha
TGF-β1	Transforming growth factor beta-1
