# Supplementary material for: Multivariate Temporal Inflammatory–Regenerative Signatures of Bovine Platelet-Rich Gel Supernatants Under Different Storage Temperatures
Source: Gels. 2026 May 12;12(5):422. doi: 10.3390/gels12050422 (PMC13205330; doi:10.3390/gels12050422)
Supplement: Supplementary file 1 [file gels-12-00422-s001.zip › Figures S1-S4.pdf]

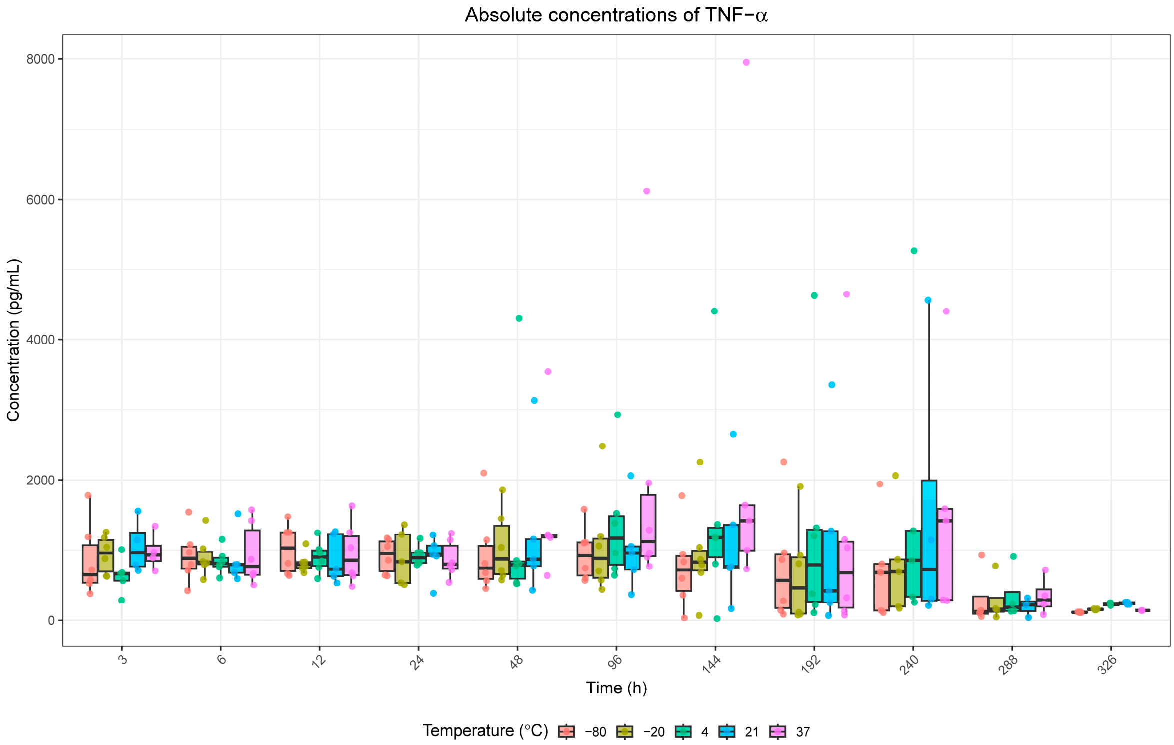

Figure S1: Box-and-whisker plots of absolute TNF-α concentrations across storage temperatures and time points, including individual data points. Boxes represent the interquartile range (IQR), the central line indicates the median, whiskers extend to the minimum and maximum non-outlier values, and dots represent individual observations.

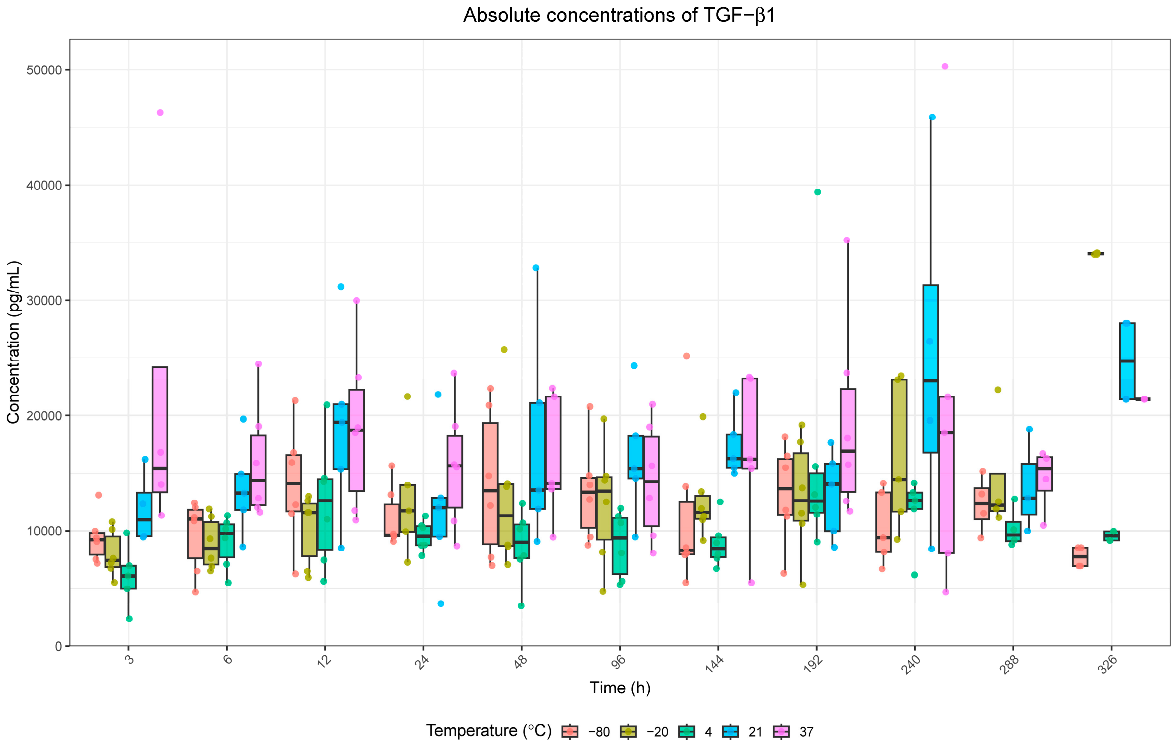

Figure S2: Box-and-whisker plots of absolute TGF-β1 concentrations across storage temperatures and time points, including individual data points. Boxes represent the interquartile range (IQR), the central line indicates the median, whiskers extend to the minimum and maximum non-outlier values, and dots represent individual observations.

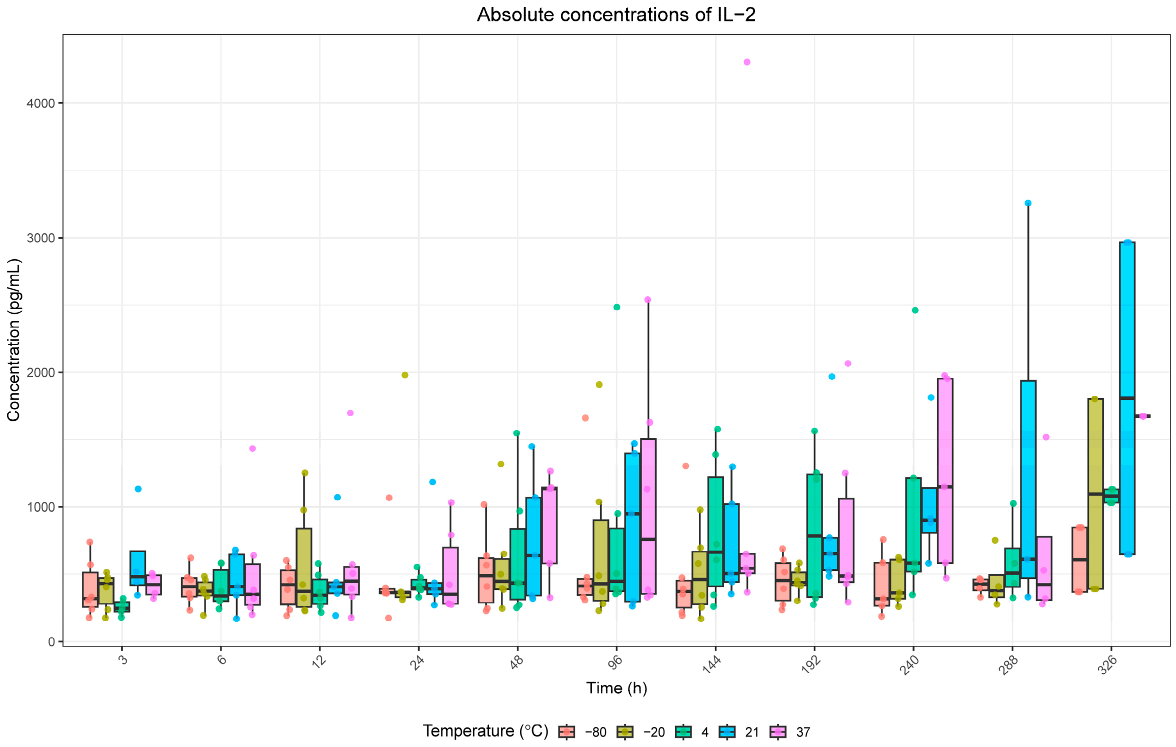

Figure S3: Box-and-whisker plots of absolute IL-2 concentrations across storage temperatures and time points, including individual data points. Boxes represent the interquartile range (IQR), the central line indicates the median, whiskers extend to the minimum and maximum non-outlier values, and dots represent individual observations.

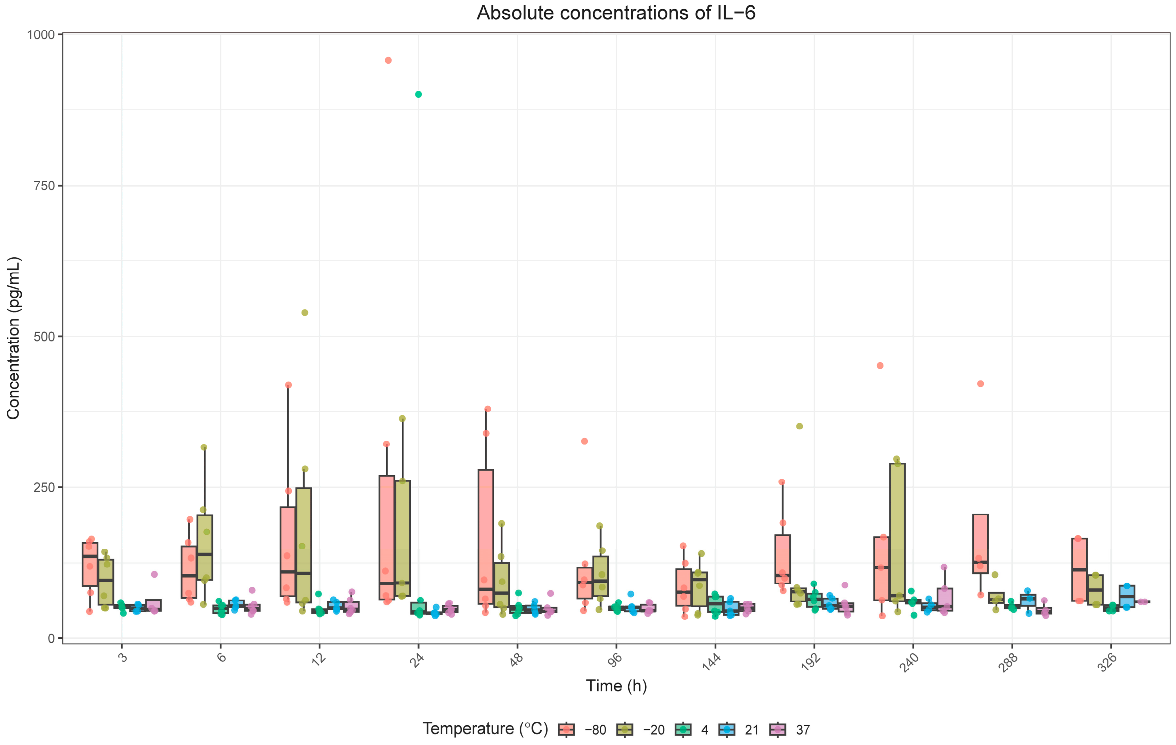

Figure S4: Box-and-whisker plots of absolute IL-6 concentrations across storage temperatures and time points, including individual data points. Boxes represent the interquartile range (IQR), the central line indicates the median, whiskers extend to the minimum and maximum non-outlier values, and dots represent individual observations.
